# Draw yourself: How culture influences drawings by children between the ages of two and fifteen

**DOI:** 10.3389/fpsyg.2022.940617

**Published:** 2022-11-08

**Authors:** Sophie Restoy, Lison Martinet, Cédric Sueur, Marie Pelé

**Affiliations:** ^1^Université de Strasbourg, CNRS, IPHC UMR 7178, Strasbourg, France; ^2^Institut Universitaire de France, Paris, France; ^3^Anthropo-Lab, ETHICS EA 7446, Université Catholique de Lille, Lille, France

**Keywords:** representation, self-portrait, cross-cultural study, drawings, child development

## Abstract

The place children live strongly influence how they develop their behavior, this is also true for pictorial expression. This study is based on 958 self-portraits drawn by children aged 2–15 years old from 35 countries across 5 continents. A total of 13 variables were extracted of each drawing allowing us to investigate the differences of individuals and environment representations in these drawings. We used a principal component analysis to understand how drawing characteristics can be combined in pictorial concepts. We analyzed the effect of age, gender, socioeconomic, and cultural factors in terms of complexity and inclusion of social (human figures) and physical (element from Nature and man-made elements) environments, their frequencies, size, and proportions of these elements on each drawing. Our results confirm the existence of cultural variations and the influence of age on self-portrait patterns. We also observed an influence of physical and socio-cultural contexts through the level of urbanization and the degree of individualism of the countries, which have affected the complexity, content and representation of human figures in the drawings studied.

## Introduction

Drawing is one of the different ways that human beings express themselves (Lange-Küttner, 2020). This was already the case thousands years ago, as shown by the Sulawesi warty pig painting found on the walls of a cave in Indonesia and dated to at least 45,500 years ago (Brumm et al., 2021). Long seen as the prerogative of modern humans, the findings of older traces cast doubt on the identification of the first species to show drawing behavior. To date, human lineage alone has the capacity to produce figurative drawings (DeLoache, 2004). In young children, drawing is a fundamental early activity as they have not mastered verbal language at this age (Wallon et al., 1998). The analysis of children’s drawings became a very active and pluridisciplinary research area from the end of the 19th century onward, and are now a central interest for many researchers who hypothesize that children’s drawing productions represent their state of mind, thus providing a doorway into their internal world (Rübeling et al., 2011).

But how should we define drawing? This term simultaneously defines an action (a behavior) and an artifact (the result of this behavior) (Kourkoulis, 2021). Drawing behavior makes it possible to represent objects, thoughts or feelings through visible graphic elements, regardless of whether they are interpretable or not (Martinet et al., 2021). Humans therefore use drawing as a projective tool that can make their internal life, perceptions and experiences visible (Ouedraogo, 2015). This “mirror of the soul” reflects the representativeness of a drawing, and results from the perspectives of both, the one who produces it and the one who observes it. Thus, the child who draws possess an internal representativeness of his or her work, while the adult who analyses the drawing only perceives its external representativeness (Martinet et al., 2021). The more similar the representativeness of both perspectives is, the more convinced we are that children world can be understood from their drawing. A drawing is therefore described as figurative when it can be read without ambiguity by others and permits communication (Kourkoulis, 2021), whatever its degree of realism (Willats, 2005). The analysis of this drawing allows us to understand certain aspects of representations by children, and namely their self-image.

In this respect, it is therefore essential to take into account the development of drawing behavior in humans (Toku, 2001; Lange-Küttner, 2008, 2014). Pictorial expression is made possible by the interaction of three ontogenic stages. The first is the improvement of the child’s motor coordination. The second is the development of perceptual skills, which increase with improved levels of attention. And finally, the third corresponds to cognitive skills, notably when the child understands the symbolic meaning of objects and establishes pictorial repertoires (Toomela, 2002). The neurological development occurring during early childhood (0–4 years) allows children to understand themselves and others, at around the age of three, and gives them the ability to communicate with the help of visual symbols (DeLoache, 2004). Children motor and cognitive development is therefore the root of the progressive complexity of their drawings.

Many researchers (Luquet, 1927; Lowenfeld and Brittain, 1987; Baldy, 2005; Royer, 2005; Marcilhacy and Demirdjian, 2011) concur that regardless of the environment in which the drawing child has grown, there is a progression that begins with scribblings, then figurative sketches and finally detailed drawings (Golomb, 1992). Toward the age of two, children begin to draw and include certain graphical elements that they seek to reproduce (Picard and Zarhbouch, 2014). These first artistic productions are referred to as *scribbles* (Kellogg, 1970). At the age of 3–4 years, the first representations of human figures appear in the form of *tadpole figures*. Subsequently, entering infant school provides children with an opportunity to draw, write and understand socially shared meanings (Cox, 1992). This training increases their drawing experience and their use of figurative signifiers (Martinet et al., 2021). Thus, children add external representativeness to their drawing from the age of 4–5 years (Baldy, 2005). Their productions then become more and more differentiated and complex with age (Golomb, 1992), before reaching a critical period at puberty (i.e., period of oppression) (Cohn, 2012) when their drawing activity ceases in favor of verbal language, which is more flexible and economical (Baldy, 2011). An important point is that this critical period as well as other stages of graphic development are not always found at the same ages or in the same way from one culture to another (Toku, 2001; Cohn, 2014).

Drawing behavior is therefore composed of an innate element–a blind individual not exposed to a graphic universe will have resilient capacities to produce a rudimentary figurative drawing (Millar, 1975; Golomb, 1992; Andersson and Andersson, 2009; Cohn, 2012)- but also has another culturally acquired component (Cohn, 2012; Picard and Zarhbouch, 2014). Indeed, the existence of the above general models does not rule out the influence of environmental factors (Rübeling et al., 2011). The recognized importance of cultural variations have changed the way drawing behavior was perceived; it would henceforth be seen in more flexible and diversified terms, some researchers going so far as to speak of graphic language (Goodnow et al., 1995; Baldy, 2011). Verbal language and graphic expression therefore share many similarities and particularly their culturally specific character (Kourkoulis, 2021). The UNESCO defines culture as a set of distinctive spiritual, material, intellectual, and emotional features that characterize a society or a social group. An organized social unit, whether familial, societal or media-based, thus holds its own understanding of the world which is transmitted throughout generations. In this way, children gradually develop a culturally informed understanding of themselves and others (Rübeling et al., 2011; Kourkoulis, 2021). The eco-cultural approach defines two major developmental strategies. In the first, each person sees themselves as part of an economically and socially interdependent whole: this is called the *interdependence strategy*. Conversely, the self is central in the second strategy, and the individuals see themselves as unique and separate from others: This is called the *independence strategy* (Keller, 2007). Children therefore have a different perception of themselves and others according to their culture (Markus and Kitayama, 1991), and this can be observed through their drawings (Rübeling et al., 2011). These two strategies will influence many human behaviors as the way pedestrians cross the road (Pelé et al., 2017), how they share rewards (Kim et al., 1990) or knowledge (Moss et al., 2007). Then, if asked to realize a self-portrait, we may hypothesize that a child from an individualistic culture will represent him/herself in bigger proportions compared to a child who grows in a more collectivist one. In this latter case, we can imagine that the child will implement other people or others elements in his/her self-portrait.

Paying attention to the influence of culture on drawings is relatively recent (Jolley et al., 2010), but some authors have confirmed the existence of cultural variations (Lindström, 2000; Cox et al., 2001; Toku, 2001; Cohn, 2014; Picard and Zarhbouch, 2014). When children draw human figures, they do not seek to represent an anatomically correct body but rather draw people (Rübeling et al., 2011) by varying the height, facial details (Gernhardt et al., 2015, 2016), and facial expressions (La Voy et al., 2001) of these representations. Studies based on qualitative data give examples of the influence of culture on young people who draw. Cox et al. (2001) observed that Japanese children draw motionless figures that are seen from the front or in profile, immobile or running, and that these drawings were of better quality than those drawn by children in the United Kingdom. The authors attribute this superiority to the influence of manga comics on Japanese children. Nomoto (2007) compares the Rey complex figures drawn by French and Japanese children and how these figures evolve over time. He found that while French children attach importance to the overall effect of their drawing and constantly improve the proportions of the figures they draw; Japanese children show meticulous attention to detail and draw increasingly detailed figures. Existing researches suggest that these trends may reflect the differences between the two education systems: The Japanese tradition encourages imitation and insists on the importance of paying attention to details, whereas French education encourages a more global and spontaneous cognition style (Cohn, 2014). The self-portrait is a good means to observe the cultural influence on drawing. Indeed, the predominant cultural model is imprinted on our sense of identity, and reflects partly the degree of attachment we have for the elements surrounding us (Kourkoulis, 2021). The presence or absence of environmental details in the drawings therefore appears to testify their value to the child (Picard and Zarhbouch, 2014).

Studies have observed the understanding of the self and of the social world from the details of drawn human figures, but few have closely examined the elements of environment (elements from Nature and man-made objects) depicted in these drawings. On the same way, only few studies focused on the influence of the level of urbanization on the drawings of children (Rübeling et al., 2011). Moreover, the number of cultures and countries considered in cross-cultural studies is also very limited. Current research mostly focuses on Joseph Henrich’s W.E.I.R.D. acronym, which designates Western, Educated, Industrialized, Rich, and Democratic societies (Henrich et al., 2010); it is therefore important to study other societies in order to obtain a clear and global picture of drawing behavior in our species. Our study sought to observe the differences in the representation of the individual and of their environments (social and physical) in self-portraits drawn by children from a large number of countries. We analyzed a total of 958 scanned and available self-portraits produced by children aged 2–15. These children were from 35 countries located on 5 different continents (Porte, 2012). In total, we measured 13 indices-based on classical studies of drawing in children (level of complexity, Baldy, 2005; representation of human figures, Rübeling et al., 2011) but also representation of non-human elements (from Nature and man-made objects)-that we implemented in a principal component analyses (PCA). PCA is used to extract and visualize important information contained in a multivariate data table by combining metrics to form a biologically or psychologically significant dimension, as already shown for personality (Wolf and Weissing, 2012; Bousquet et al., 2015), sociality (Viblanc et al., 2016) or even for drawing (Sueur et al., 2021). This method is expected to combine indices in dimensions linked to important psychological or cultural concepts as the self-representation, the family, or the importance of elements from Nature or man-made objects. We expected that these dimensions will be directly linked to the socioeconomic and cultural environment of children. We also expected drawings to be exclusively self-portraits in individualistic societies (i.e., independence strategy) and to be opened to the family or to elements of Nature in collectivist ones (i.e., interdependence strategy). Children from urban areas may have access to a more graphical environment (architecture, advertising, etc.) which can contribute to the way they represent themselves. Considering that self-representation develops from scribbling to the tadpole figure and finally to more detailed and realistic drawings, we also sought to determine whether any aspects of the drawings could be associated with the age and the gender of the children.

## Materials and methods

### Collection of drawings

**FIGURE 1:**
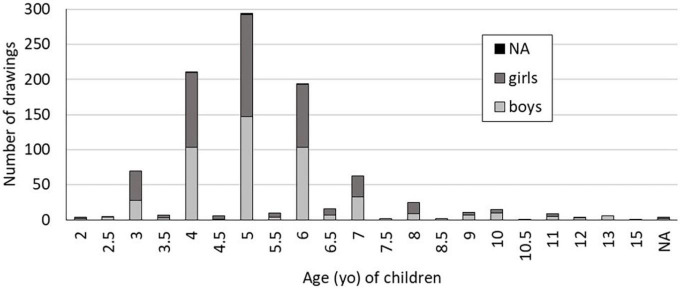
Numbers of drawings according the age and gender of children.

The drawings are 958 self-portraits by children (468 girls; 475 boys; 15 not stated) aged 2–15, from 35 countries spread over 5 continents. In each country, drawings were collected in one to four towns (details given in Supplementary Table 1). They are taken from the Early Pictures online archive and were collected between 2005 and 2012 by the French photographer Gilles Porte^1^. Analysis concerns only one drawing per child. The drawings presented by Gilles Porte were produced according to the following procedure: Children were given a sheet of black paper in format A5 and a white pencil (crayon). The crayon was prepared (sharpened) in advance. Children were asked to position the paper in “portrait” format when drawing. After such preparation, the children were asked to draw themselves. No further comment was given, and the time of drawing was free. When they finished, they gave back the papers, and names and ages were indicated on the back of drawings by Gilles Porte and the adults in charge on site. The satisfactory balance of this database allowed us to consider both age and gender of children (Figure 1).

### Ethical note

Children’s participation in the creation of this database was on a voluntary basis and was subject to school approval and parental consent. The photographer, Gilles Porte, was given help and permission from Non-Governmental Organizations and the UNESCO to approach certain populations. We have respected the license of this database insofar which was strictly used for research purposes at the Hubert Curien Pluridisciplinary Institute (CNRS), a state-recognized research institute. The database was solely used to discuss the pictorial development of children, with no individual psychological or psychoanalytic interpretation. The ethical rules of this database have therefore been respected.

### Drawing analysis

All measures were made by SR and double-checked by MP following ethological sampling (Pelé et al., 2021; Sueur et al., 2021). For each drawing, the following stages of analysis were performed.

#### Interpretation of the drawing

Each drawing was coded as *figurative* if it had an unambiguously recognizable external representation, *non-figurative* if no graphic element could be interpreted by an outside observer, or *mixed* if it contained both figurative and non-figurative elements.

As previously said, children can represent elements or people that are valuable for them in their drawings (Picard and Zarhbouch, 2014; Kourkoulis, 2021). So, in figurative and mixed drawings, we specifically looked at representation of human figures but also at non-human elements (Nature, man-made objects, and symbols).

#### Representation of human figures

**FIGURE 2:**
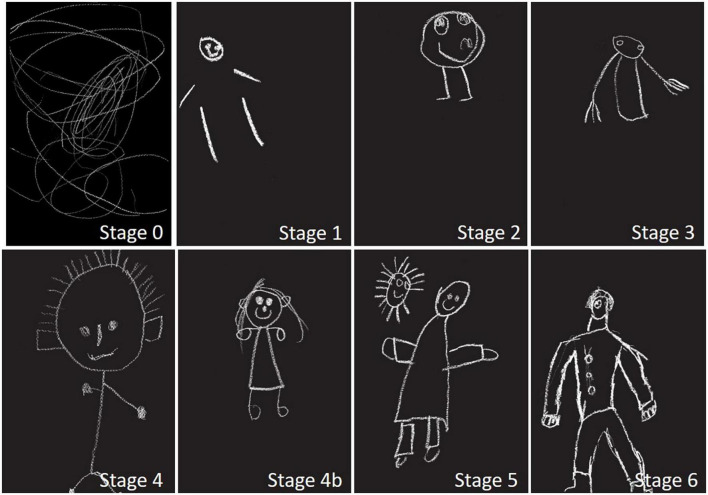
Examples of the different types of human figures examined in our study.

Concerning the representations of human figures, we first used a recognized classification model that could determine their complexity. This is known as Baldy classification (Baldy, 2005), which defines six stages of transformation of the morphology of the human figure: The round and enumerated human figure (stage 1), the tadpole (stage 2), the intermediary (stage 3), the conventional man that is first filiform (stage 4) then tube-shaped man (stage 5), and finally the outline (stage 6). A mixed intermediate stage (stage 4b) has been added here to define the men composed of single and double lines. A lower (stage 0) has been created for non-figurative level drawings (Figure 2).

**FIGURE 3:**
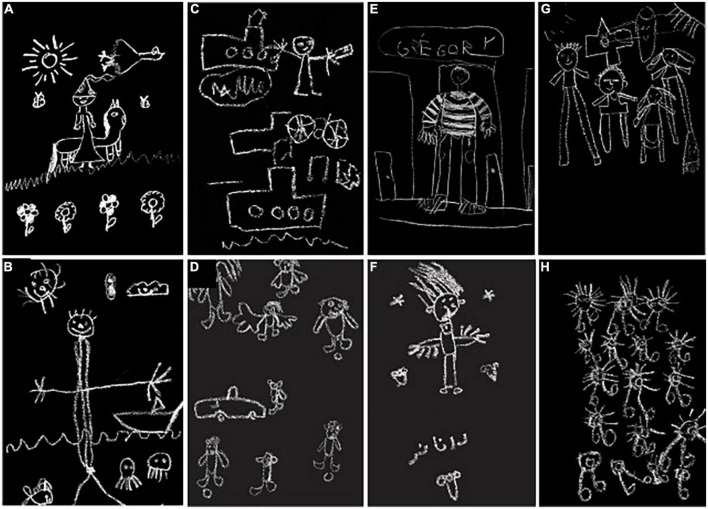
Examples of drawings. Four types of non-human figures have been considered: elements representing nature (e.g., tree, flowers, sun; **A,B**), man-made objects (e.g., house, car, and boat, **B–E,G**), symbols **(F)**, and characters (i.e., letters or numbers, **E**).

As the proportion and the level of details can differ from culture to another (Cox et al., 2001; Nomoto, 2007; Cohn, 2014), we then noted the number of human figures present in each drawing (for one individual, see Figures 3A–C,E,F; for several individuals see Figures 3D,G,H) and the presence (and the number) of sensory organs in the human figures (eyes, nose, mouth, ear, and hand).

Considering proportion of the human figures in the drawings, we measured the size of each head and body drawn by children using the GIMP 2.10.22 software (Peck, 2006). The precision of measurements was improved through the use of a 10 × 10 grid (Casti, 2016) as well as compass and selection tools. GIMP was also used to determine the minimum convex polygons (MCP) which is the smallest polygon that can be drawn around the extreme location points, and which has angles that all measure less than 180 degrees. Commonly used to estimate the home range of animals (Nilsen et al., 2008), MCP was calculated here to measure the coverage of the human figure on the sheet. MCP varies from 0 (no drawing at all) to 100% (the drawing covered the entire sheet). All of these analyses allowed us to add four new indices from our raw data, namely the complexity of the face, i.e., the sum of the observed facial elements ranging from 0 to 5; the proportion of the head to the body size for drawings of human figures (head-to-body ratio in centimeters); the size of drawings and the size of human figures as a percentage of the drawing sheet’s coverage. We achieved this by dividing the minimum convex polygon by the total number of pixels of the sheet.

#### Representation of non-human elements

Four types of non-human figures have been considered: Elements representing Nature (e.g., tree, flowers, and sun; see Figures 3A,B), man-made objects (e.g., house, car, boat, and figure; see Figures 3B–E,G), symbols (e.g., heart and stars, see Figure 3F), and characters (i.e., letters or numbers, see Figure 3E). For each of these categories, the number of elements present in each drawing have been noted and since these different elements can be found on the same drawing, they are not exclusive. We also observed whether these drawings included a personification of Nature by noting the presence of facial details on the natural elements represented (see Figures 3B,G).

At the end of drawing analyses, a total of 13 variables were analyzed for each drawing, namely: (1) Its level of complexity [based on Baldy’s (2005) classification] and (2) its type (*figurative*, non-*figurative*, and *mixed*); the presence of (3) human faces and hands, (4) elements from Nature and (5) man-made objects, and (6) the personification of 4 and 5; (7) representations of animals, (8) additional human figures, (9) symbols, (10) letters and numbers, and finally the size of (11) head of human figure (ratio head/body), (12) human figures (MCP), and (13) entire drawings (MCP) (Table 1).

**TABLE 1:** Metric loading for the five PCA dimensions of our data.

Variables	Dim. 1	Dim. 2	Dim. 3	Dim. 4	Dim. 5
(1) Level of complexity (from Baldy, 2005)	**0.81**	–0.317	0.053	–0.045	–0.03
(2) Type of drawing (*figurative, non-figurative*, and *mixed*)	**0.826**	–0.267	–0.165	0.048	–0.082
(3) Presence of human faces and hands	**0.752**	–0.299	0.051	–0.009	0.16
(4) Presence of natural elements	0.317	**0.568**	–0.26	0.01	–0.017
(5) Presence of man-made objects	0.168	0.377	–0.082	0.122	−**0.503**
(6) Personification of elements 4 and 5	0.306	**0.525**	–0.278	–0.258	0.239
(7) Presence of animals	0.264	**0.501**	0.342	–0.24	0.176
(8) Additional human figures	–0.07	0.2	**0.625**	–0.283	–0.315
(9) Presence of symbols	0.139	0.196	0.065	**0.632**	0.271
(10) Presence of letters and numbers	0.135	0.133	0.131	**0.737**	–0.145
(11) Head/body ratio (in cm)	–0.154	0.055	0.303	0.043	**0.687**
(12) Size of the human figures (when present) (MCP)	0.412	0.079	**0.759**	–0.16	0.073
(13) Size of the entire drawing (MCP)	0.122	**0.799**	0.315	0.054	–0.016

Colored cells and boldness values indicate the dimension in which each variable was retained. Loading represents the correlation or importance of representativity of a variable in a dimension.

TA positive (respectively, negative) loading indicates a positive (respectively, negative) correlation between a variable and a dimension.

Bold and gray highlighted cases indicate loading for which variable were retained for a dimension, based on the highest value.

### Specific indices of culture

As our study aimed to observe the influence of socioeconomic and cultural conditions on drawing behavior of children, data for three specific indices were collected to categorize each country. The first is the Individualism Index Value (IDV), which assesses the links between individuals and the members of their community as a degree measured on a scale of 0–100. A high IDV value indicates that the society can be considered more individualistic (i.e., an independent strategy), while a low value indicates a more community-based society (i.e., an interdependent strategy) (for details about factors included into this metric, see Hofstede, 2010). The second is the Urban Development Index (UDI) which corresponds to the percentage of the population living in urban areas compared to the total population of a country (Molinaro et al., 2020). Indeed, a child living in urban areas has access to a more graphical environment (architecture, advertising, etc.) which can contribute to the way he or she will draw and represent him/herself. However, a child in a largely rural country could have been recruited in a large city, while a child from a largely urban country from a small village whilst children were recruited in schools for which we can assume that they were not only passing through. Since urbanization of a country is not dichotomous but better corresponds to a scale we also and lastly consider the Inequality-Adjusted Human Development Index (IHDI). This index was developed by the United-Nations Development Program and reflects life expectancy, level of education and standard of living (access to culture, goods, services, and transport), and takes the country’s inequalities into account.

**FIGURE 4:**
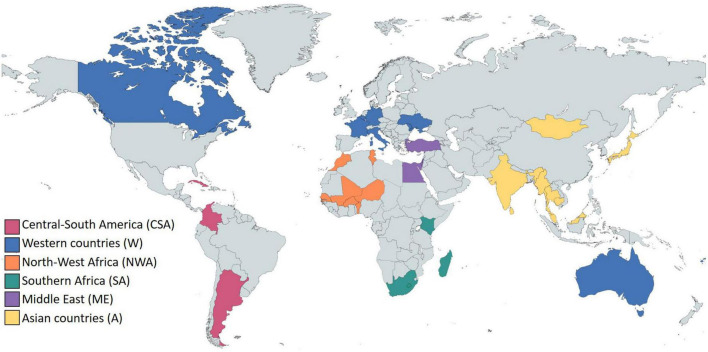
World map showing the countries and cultures studied.

In order to facilitate our data analysis and observe the influence of the main cultural trends, we split the 35 countries into six groups of culturally similar countries (CSC): Central and South America (CSA), Western countries (W), North and West Africa (NWA), Southern Africa (SA), Middle East (ME), and Asian countries (A) (Figure 4).

### Statistical analyses

Once collected for each drawing, our 13 variables were grouped into interpretable dimensions *via* principal component analysis (PCA) performed with the R *FactoMineR* package (Lê et al., 2008). PCA is used to extract and visualize important information contained in a multivariate data table by combining metrics to form a biologically or psychologically significant dimension. Here, we aimed and expected metrics to be combined and form dimensions that correspond to cultural (CSC groups) or biological (i.e., age and gender) aspects of the drawing and show performance or aestheticism in the drawing (Dissanayake, 2001; Matthews, 2003). The dimensions obtained were used as response variables, and the coordinates of each drawing in each dimension allowed us to compare the drawings according to the age, gender, IHDI-IDV-UDI, and CSC group of the children. For this, we removed some missing values of our data set (27 drawings from Roma community because the living country of the children were undetermined, four drawings for which gender was not known, and four drawings for which age was not known).

In order to test the potential effect of our multiple independent variables (gender-age-CSCgroup-IHDI-IDV-UDI), we performed a multivariate linear regression model for each dimension of our PCA using the *lm()* function from the R *car* package (Fox and Weisberg, 2019). The potential collinearity between our predictor variables was tested by calculating the variance inflation factor (VIF) of the *car* package (Fox and Weisberg, 2019). This calculation enabled us to remove the IHDI variable that was too strongly correlated with the CSC group variable (VIF > 11) and confirm the absence of problematic (multi) collinearity with other variables (VIF < 4). Our models thus included two factors, namely gender (2 levels) and CSC group (6 levels), and 3 numerical variables, i.e., age, IDV, and UDI. Interaction between age and gender was tested but was not found to be significant and was therefore excluded from the model (0.3 < *p* < 0.99). Given the absence of a normal distribution and homogeneity in our values, we decided to carry out non-parametric tests. The preconditions for these tests were met by our numeric dependent variables, our balanced samples and weakly correlated predictor variables. The *p*-value was then calculated by sampling, performing a Monte-Carlo test with 1,000 permutations for each model with the *PermTest()* of the R *pgirmess* package (Giraudoux et al., 2018). Tests were run three times to check the statistical stability. The significant factors in our models were then observed more closely using *post-hoc* pairwise comparison with the *pairwisePermutationTest()* function of the R *rcompanion* package (Mangiafico, 2019). Consequently, a Benjamini–Hochberg correction was applied. Finally, we observed the force and direction of the correlations between the significant numerical variables and the PCA dimensions.

All the statistical models were carried out using the R software, version 4.0.2 (R Core Team, 2013) with α = 0.05.

## Results

Out of a total *n* = 958 drawings analyzed, 33% showed environmental elements as well as the self-portrait. Amongst these, 54% contained natural elements (9.5% of which had been personified), 12% showed animals, 13.5% man-made objects, 10% supplementary human figures, 4% symbols, et 3% letters or numbers. On average, the children drew on 35 ± 20% of the total paper sheet. Human figures took up an average 16 ± 20% of the drawing space and 87% of these figures included facial elements, with an average of 2.9 ± 1.5 facial details included per drawing.

Five dimensions were retained from the 13 variables for our PCA, with eigenvalues of at least 1. These dimensions explained 63% of the total data variance (dimension 1 = 18.8%, dimension 2 = 15.4%, dimension 3 = 11.4%, dimension 4 = 9.2%, and dimension 5 = 8.1%). Each metric showed a higher loading (*r* > 0.5) in one dimension compared to the others, thus enabling us to classify them (see Table 1).

Dimensions 1, 2, 3, and 4 show significant variables to which they are positively correlated, while the fifth dimension is positively correlated with the head-to-body ratio but is negatively correlated with the number of man-made objects. We were therefore able to determine that the first PCA dimension represented the complexity of the drawing but also the meaning, the second was characterized by the inclusion of the living environment, the third corresponded to the space attributed to human representation, the fourth represented the characters grouping together the symbols, letters and numbers, and finally the fifth dimension showed the construction of identity, i.e., the importance that children attribute to what they are, built on their own experience and in relation with others, or at least the relative importance of the self and of objects. Of course, this interpretation of the dimensions are quite subjective and we come up to this later in the discussion.

**FIGURE 5:**
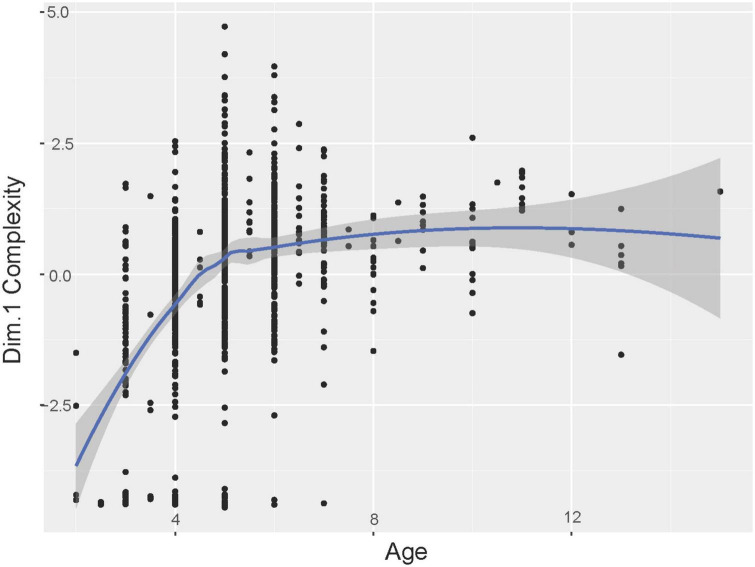
Children’s drawing behavior for dimension 1 (i.e., complexity) according to age.

Following the removal of the 35 missing value lines from our dataset, *n* = 923 drawings were considered in our analyses based on the use of 10 variables (i.e., the five PCA dimensions; the gender; the age; the CSC group; the IDV; and the UDI). Three prediction variables were found to significantly influence the values of dimension 1: Age, CSC group, and the UDI (gender *p* = 0.129; age *p* < 0.001; culture *p* < 0.001; IDV *p* = 0.885; and UDI *p* = 0.024). However, pairwise comparisons revealed that there was no significant difference between countries groups (*p* > 0.5). We also observed that the complexity of the drawings increased with the age of the children (*t* = 11.81; *R*^2^ = 0.130; *r* = 0.361) and to a lesser extent with the UDI (*t* = 2.278; *R*^2^ = 0.004; *r* = 0.067) (Figure 5).

**FIGURE 6:**
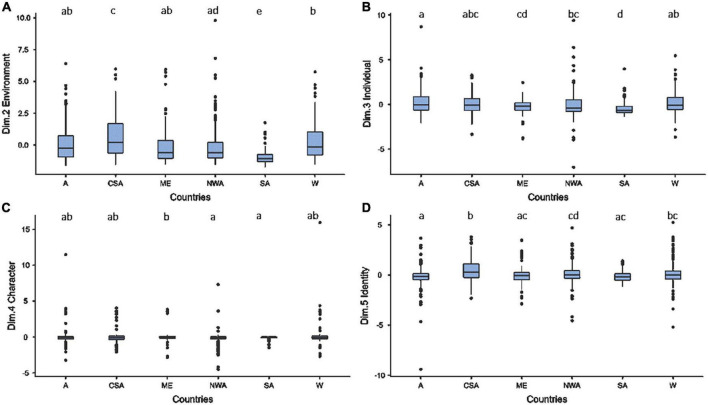
Comparison of children’s drawing behavior for **(A)** the dimension 2 (i.e., environment), **(B)** the dimension 3 (i.e., individual), **(C)** the dimension 4 (i.e., character) and **(D)** the dimension 5 (i.e., identity). The countries groups that have no letters in common differ significantly.

Two variables were significant in the second dimension (gender *p* = 0.053; age *p* < 0.001; culture *p* < 0.001; IDV *p* = 0.946; and UDI *p* = 0.887). These two variables are age and CSC group. *Post-hoc* comparisons revealed ten significant differences among pairwise comparisons between CSC groups (Table 2). More specifically, children from Central and South America showed more elements of the environment in their drawings than those from other cultures. Drawings by children from Western countries also included elements of their environment more frequently than those drawn by children from African countries and the Middle East. We also note that children living in Southern Africa tend to include fewer elements of the environment in their work than children from countries in North and West Africa, the Middle East and Asia (Figure 6A). The tendency to include elements of the environment decreases with the age of the children (*t* = −6.423; *R*^2^ = 0.041; *r* = 0.204).

**TABLE 2:** Adjusted *p* of pairwise cultural similar countries (CSC) comparisons for dimensions 2–5 (*p < 0.05; **p < 0.01; and ***p < 0.001).

Countries	Dim. 2 environment	Dim. 3 individual	Dim. 4 character	Dim. 5 identity
A vs. CSA	< 0.001***	0.247	0.596	< 0.001***
A vs. NWA	0.263	0.042*	0.343	0.019*
A vs. SA	< 0.001***	< 0.001***	0.575	0.587
A vs. W	0.242	0.336	0.363	0.006**
A vs. ME	0.645	< 0.001***	0.076	0.421
CSA vs. NWA	< 0.001***	0.626	0.318	0.017*
CSA vs. SA	< 0.001***	0.008**	0.333	< 0.001***
CSA vs. W	0.007**	0.575	0.783	0.073
CSA vs. ME	0.002**	0.079	0.343	0.002**
NWA vs. SA	< 0.001***	0.042*	0.616	0.081
NWA vs. W	0.04*	0.241	0.124	0.587
NWA vs. ME	0.645	0.241	0.011*	0.28
SA vs. W	< 0.001***	< 0.001***	0.329	0.046*
SA vs. ME	< 0.001***	0.273	0.017*	0.587
W vs. ME	0.294	0.004**	0.385	0.146

Dimension 1 is not present as not influenced by the CSC variable.

CSA, Central and South America; W, Western countries; NWA, North and West Africa; SA, Southern Africa; ME, Middle East; and A, Asian countries.

Three variables stand out significantly for the third dimension, namely age, CSC group, and IDV (gender *p* = 0.954; age *p* < 0.001; culture *p* < 0.001; IDV *p* < 0.001; and UDI *p* = 0.907). *Post-hoc* comparisons revealed seven significant differences between CSC groups (Table 2). As a result, we note that the space attributed to humans in children’s drawings was significantly larger in Asia than in African countries and the Middle East. In addition, children from Southern Africa used less space for human representations than children from Central and South America, North and West Africa or–above all–Western countries (Figure 6B). It was also evident that the space attributed to human representations tended to decrease with age (*t* = −3.40; *R*^2^ = 0.011; *r* = 0.106). Conversely, the amount of space increased with the IDV of the countries (*t* = 3.683; *R*^2^ = 0.013; *r* = 0.115).

In the fourth dimension, two variables were significant, namely CSC group and UDI (gender *p* = 0.084; age *p* = 0.415; culture *p* = 0.005; IDV *p* = 0.688; and UDI *p* = 0.042). *Post-hoc* comparisons revealed significant differences between three CSC groups (Table 2). Children from the Middle East included significantly more characters in their drawing (0.3 ± 1.2) than Southern African (−0.1 ± 0.2) and North and West African (0.1 ± 0.9) children did (Figure 6C). The inclusion of characters also increases with the UDI (*t* = 3.127; *R*^2^ = 0.009; *r* = 0.097).

Finally, the fifth dimension had three significant variables: CSC group, IDV, and UDI (gender *p* = 0.689; age *p* = 0.739; culture *p* < 0.001; IDV *p* = 0.008; and UDI *p* = 0.002). *Post-hoc* comparisons then revealed seven significant differences among CSC groups when compared two by two (Table 2). For example, Central and South American children drew human figures with larger heads in relation to their bodies and included fewer man-made objects in their drawings than children in all other cultures except those of Western countries. Children in Western societies drew proportionately larger heads and fewer man-made objects than Asian and Southern African children. This tendency was higher in children in African countries than those in Asian countries (Figure 6D). Our analyses also showed that the UDI (*t* = 5.464; *R*^2^ = 0.030; *r* = 0.097) and the IDV (*t* = 2.907; *R*^2^ = 0.008; *r* = 0.895) were positively correlated with identity construction.

Our results do not indicate any significant effect of child gender on the specific drawing variables studied.

## Discussion

Our study sought to verify the existence of cultural differences in graphical representations of the self and the social and physical environments, drawn by children aged 2–15 years and originating from different countries. Using a Principal Component Analysis, self-portraits drawn by the children were evaluated in terms of their complexity and meaning, the frequency with which children included elements of their environment, the proportions used to draw human figures and the space attributed to it in terms of the self (i.e., individualism) or the presence of objects (i.e., materialism) and finally the presence or absence of characters (i.e., letters, numbers, and symbols). This is the first time that pictorial elements described in many previous studies were analyzed and combined in this way, revealing patterns and structures that could be universally studied in drawings. The cultural differences that we observed in these self-portraits confirmed that drawing is partly an acquired behavior that is widespread in humans (Kourkoulis, 2021). The physical and sociocultural environments of children appear to shape many aspects of the graphical representations they produce.

Firstly, there was no significant difference in drawing complexity between the different cultures; we could therefore note a worldwide characteristic for the development of human figure composition, with the presence of general models that evolve as the child grows older (Golomb, 1992). The observation of similar scribbles in all the CSC groups evaluated suggests that this expression is a universal step in the development of graphism (Baldy, 2009). Indeed, cognitive and motor capacities must be developed before children can create representations; graphic complexity including the meaning of the production is therefore positively correlated with the growth of the child (Toomela, 2002). However, the influence of culture on this complexity strengthens the hypothesis that the acquisition of graphic shapes and skills differs according to each child’s cultural environment. Contrary to certain studies, we have not noted an earlier development of complexity in drawings produced by Asian children who generally produce more detailed graphic compositions through the sustained learning of drawing behavior in their education systems and the common presence of manga drawings in their societies (La Voy et al., 2001). To remind, the Inequality-Adjusted Human Development Index (IHDI) we removed from analyses because of collinearity is linked with the CSC groups as with the urbanization level.

The urbanization level of children’s living environments also affects their graphical production in different ways. Our findings show that children from urbanized environments produced more complex drawings with more facial details (Gernhardt et al., 2015) that are even present in their very first representations of human figures (Rübeling et al., 2011). The influence of urbanization is explained by the child’s access to drawing materials and opportunities, in particular through the teaching of art at school, and the wide availability of graphical models that children can copy, particularly through the media. A child’s graphical capacity is therefore likely to depend on the stimulation provided by their environment, which defines their experience of the pictorial world (Picard and Zarhbouch, 2014). Our findings also suggest that children in urban societies include characters (symbols, numbers, and letters) more frequently in their drawing. As education level is linked to urbanization, our result may be due to the wider presence of educational structures in urbanized societies, thus providing a favorable environment for the learning of verbal language and writing that are then reflected in the child’s drawings. Linguistic differences could also explain the presence or absence of symbols or letters in drawings, because the complexity of certain native languages requires a faster evolution of the cognitive capacities that children also use to draw (Toku, 2001). Children who do not attend school and live in social environments with sparse sociocultural means have very limited graphical language and poor levels of inventiveness. Children who attend school and live in areas that are rich in sociocultural and artistic models develops a rich graphical language that facilitates creation. Finally, we observed that the level of urbanization also influences the construction of identity in children: In more urban societies, they are more likely to over-represent themselves in their drawings, with a larger head and the absence of man-made elements. Intercultural studies and the few available historical comparisons globally suggest that diverse cultural environments may provide different evolutive pathways leading to a wide range of drawing types (Baldy, 2009). Children from villages in developing countries are not “behind” Western children in terms of graphical development, but develop distinct graphical styles derived from different cultural patterns. They are on different developmental paths. As highlighted by Merleau-Ponty (2001), what we consider to be the “normal” endpoint of graphical development is actually one of many possible cultural achievements.

These parameters are also influenced by the degree of individualism inherent to each culture. Indeed, societies promoting individualism lead children from childhood toward independence and autonomy, both of which require the early development of confidence and self-esteem (Keller, 2007). This self-esteem is associated with a decrease in materialistic values in childhood (Chaplin and John, 2007), thus influencing graphical representations. Our results show that this individualism also influences the size and number of human representations in the drawings, thus confirming the conclusions of previous studies (Rübeling et al., 2011). The more individualistic a society is, the greater the child’s perception of his own value within his culture will be and the more he will maximize his existence as an individual, in particular by representing himself graphically as a large figure that is alone on the paper (La Voy et al., 2001). The space attributed to human representations appears to decrease as the children grow older. This change could be explained by the increased precision and dexterity of graphical representations as the individual gets older, thus making possible to successfully produce smaller representations (Cohn, 2012).

Unlike some authors (Burkitt et al., 2004), we observed that the tendency to include environmental elements in self-portraits decreased with age. We believe that children’s school and social experiences could gradually encourage the standardization of representations (Toku, 2001). As they grow older, children may therefore seek to shape their drawing to what their cultural model defines as a “good” representation, thus reducing the number of elements they include in their drawing (Rübeling et al., 2011). Gilles Porte’s instruction to “*Draw yourself*” could also encourage children to use less elements to draw themselves (Martinet et al., 2021): The more detailed the instructions are, the more specific the drawings will be (Smith, 1993). Older children may therefore focus more on the instruction due to the evolution of their attention capacity and ability to retain information (Sutton and Rose, 1998), thus leading them to include fewer environmental elements in their drawings (Toomela, 2002). American, European and Asian children seem to give more importance to depicting living elements of their environment in their pictures. This brings us back to the idea that it is more common to see the personification of environmental elements in some cultures than in others (Court, 1992) but, in contrast to other studies, Asian children do not seem to depict more contextual elements than their American and European counterparts in this study (Masuda et al., 2008). Contrary to our expectations, we did not see evidence of a greater consideration of family or Nature in any of the self-portraits produced by children in collectivist societies. As the education level is linked to the CSC groups, our result may be explained by factors such as the exclusion of nature in educational practices (Toku, 2001), the influence of the media presenting more details and backgrounds in some cultures (Kourkoulis, 2021) or the imitation of traditional aesthetic styles of representation of the environment (Masuda et al., 2008). Contrary to other studies (Iijima et al., 2001; Lange-Küttner, 2011; Lange-Küttner and Ebersbach, 2013; Bozzato and Longobardi, 2021; Bozzato et al., 2021), the gender of children does not appear to influence their drawings of self-portrait. However, it is difficult to demonstrate gender-related differences, and a more thorough analysis of further aspects and types of environmental details in drawings could provide us with a clearer picture.

The preliminary results of our study confirm that to understand drawing behavior, it is both possible and necessary to take its context of emergence into account in order to understand the various cultural influences that affect it. Also, we have shown that it is possible to observe an indication of a child’s sense of identity in their drawings through the importance they assign to representations of the elements within their environment. It is, however, important to note that without data specifying what the child intended to draw, our understanding of their drawing remains subjective. Although not completely reliable on its own, the child’s verbally expressed intention can be paralleled by what the observer perceives and allows the experimenter to get a more accurate picture of the child’s drawing (Martinet et al., 2021). Additionally, our choice to group the 35 countries together to observe the major cultural influences ruled out the possibility of considering the diversity of cultural practices and educational methods between countries of the same group and between regions of the same country, which could affect drawing behavior differently. It would therefore be interesting to make more specific comparisons. The dataset being limited for some age and countries categories, it should be also important to increase the number of drawings, per age, per country but also per cities vs. villages to increase the statistical power of analyses and so the rigor of our explanations. Continuing our research would allow us to discover the wealth of information contained in children’s works in greater depth. Our methodology could be applied to examine psychological and emotional traits measured through questionnaire (Kallitsoglou et al., 2021) or direct observation (Shiakou, 2012).

In his masterpiece *The Little Prince*, Antoine de Saint-Exupéry stated that adults never understand anything by themselves, and that children always have to explain to them. What would happen if adults took their turn and tried to understand the messages children convey, by pushing the limits of their own vision through the observation of the child’s culturally specific graphical productions? From this perspective, the analysis of drawings is essential and could allow the development of new communication strategies, particularly in our world of constantly evolving cultural diversity.

## Data availability statement

The raw data supporting the conclusions of this article will be made available by the authors, without undue reservation.

## Ethics statement

The studies involving human participants were reviewed and approved by Strasbourg University Research Ethics Committee (Unistra/CER/2019-11). Written informed consent to participate in this study was provided by the participants’ legal guardian/next of kin.

## Author contributions

MP and CS contributed to conception and design of the study. SR and MP organized the database. SR and CS performed the statistical analysis. SR wrote the first draft of the manuscript. All authors wrote sections of the manuscript and contributed to manuscript revision, read, and approved the submitted version.

## Footnotes

1
https://www.early-pictures.ch/porte1/archive/en/
